# A survey about methods dedicated to epistasis detection

**DOI:** 10.3389/fgene.2015.00285

**Published:** 2015-09-10

**Authors:** Clément Niel, Christine Sinoquet, Christian Dina, Ghislain Rocheleau

**Affiliations:** ^1^Computer Science Institute of Nantes-Atlantic (Lina), Centre National de la Recherche Scientifique UMR 6241, Ecole Polytechnique de l'Université de NantesNantes, France; ^2^Computer Science Institute of Nantes-Atlantic (Lina), Centre National de la Recherche Scientifique UMR 6241, University of NantesNantes, France; ^3^Institut du Thorax, Institut National de la Santé et de la Recherche Médicale UMR 1087, Centre National de la Recherche Scientifique UMR 6291, University of NantesNantes, France; ^4^European Genomic Institute for Diabetes FR3508, Centre National de la Recherche Scientifique UMR 8199, Lille 2 UniversityLille, France

**Keywords:** epistasis detection, genome-wide association study, complex disease, biological data mining, feature selection

## Abstract

During the past decade, findings of genome-wide association studies (GWAS) improved our knowledge and understanding of disease genetics. To date, thousands of SNPs have been associated with diseases and other complex traits. Statistical analysis typically looks for association between a phenotype and a SNP taken individually via single-locus tests. However, geneticists admit this is an oversimplified approach to tackle the complexity of underlying biological mechanisms. Interaction between SNPs, namely epistasis, must be considered. Unfortunately, epistasis detection gives rise to analytic challenges since analyzing every SNP combination is at present impractical at a genome-wide scale. In this review, we will present the main strategies recently proposed to detect epistatic interactions, along with their operating principle. Some of these methods are exhaustive, such as multifactor dimensionality reduction, likelihood ratio-based tests or receiver operating characteristic curve analysis; some are non-exhaustive, such as machine learning techniques (random forests, Bayesian networks) or combinatorial optimization approaches (ant colony optimization, computational evolution system).

## Introduction

Genome-wide association studies (GWAS) have generated huge datasets in the past 8 years in order to find association between genetic polymorphisms and phenotypes. Individual risk prediction based on those discoveries was promising. Nevertheless, genetic architecture of complex diseases, such as type II diabetes, is still largely misunderstood (Vassy et al., 2014). Indeed, gene-environment and gene-gene interactions must be considered to better understand etiology of such phenotypes. In other words, various joint effects of genetic variations, namely epistasis, are likely to partly determine the disease state (Mackay and Moore, 2014). While common genome-wide association analysis checks for potential SNP-disease associations in a one-SNP-at-a-time fashion, looking for all potential epistatic interactions in such datasets will quickly result in combinatorial overload. This is why classical GWAS often left behind the daunting task of epistasis detection.

Several strategies came up to overcome the epistasis intricacy. After a first section dealing with epistasis generalities, we will present in this review the main categories of methods dedicated to epistasis detection. These methods are classified as follows. First, some exhaustive approaches for searching significant genetic marker combinations will be introduced. As some of these, like Multi-Dimensional Reduction (MDR), are not manageable at a genome-wide scale, we will next turn our attention to filtering strategies which aim at reducing the size of the dataset, thereby decreasing the size of the search space. A final section will deal with machine learning and data mining techniques. This review does not intend to provide an exhaustive list of all software programs designed to find epistatic interactions, but rather to give an overview of the main categories of strategies put forward in the last 5 years.

## Background—epistasis

During the past decade GWAS have played a central role in the discovery of genotype-phenotype associations. In GWAS analyses, geneticists rely on DNA polymorphism markers to detect these associations. One of the most popular classes of genetic markers, Single Nucleotide Polymorphism (SNP), allows comparison of allelic frequencies between a sample of cases ascertained for a disease and a sample of controls. In the standard approach, SNPs are tested one by one for statistical association with the disease (Hirschhorn, 2009). Genetic variants are considered to have independent effects on the phenotype. As a result, only additive effects are considered under this approach. This kind of analysis has been widely used for years, but results are often not as appealing as expected. Indeed, with the “one locus at a time” strategy, only a little part of the genetic variance explains the phenotype, the remaining part being referred to “missing heritability” (Maher, 2008; Manolio et al., 2009).

**Figure 1:**
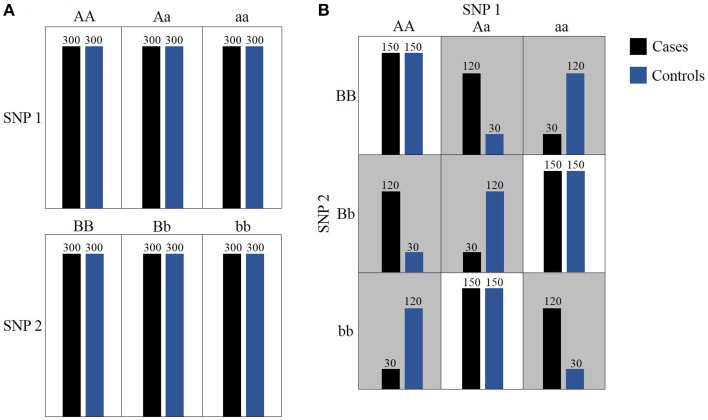
**Toy example of epistasis**. **(A)** Neither SNP 1 nor SNP 2 presents a marginal effect. **(B)** In gray cells, allele combinations between SNP 1 and SNP 2 induce statistically significant epistatic effect on the phenotype distribution.

It has been commonly admitted that missing heritability is partly due to genetic variants showing effects when they interact with one or more other variants (Eichler et al., 2010). Epistasis refers to the combinatorial effect of one or more genetic variants (Figure 1). These effects might interactively contribute besides existing marginal effects or they can also exist in absence of any marginal effect. In the last case, traditional statistical parametric methods will likely miss those interactions owing to the inflexibility of parametric models (Culverhouse et al., 2002; McKinney et al., 2006). For instance, in complex diseases like asthma (Howard et al., 2002), diabetes (Cho et al., 2004) or hypertension, additive genetic variation involves many SNPs, among which a vast majority have very small effect sizes (odds ratio less than 1.2, see Box 1) (Ritchie, 2015). As complex traits are poorly explained by additive models, one expects gene-environment or gene-gene interactions to substantially contribute to the genetics of these diseases.

Box 1 **Logistic regression and odds ratios.**

A logistic regression model is a statistical model that depicts the relationship between a linear combination of variables (e.g., SNPs in a GWAS) and a binary trait, the disease phenotype (i.e., affected/unaffected status). The probability *p* of being affected is expressed in the log scale as:

$$\begin{array}{l} \log\left(\frac{p}{1-p}\right)=\alpha \;+\;\beta_{1}x_{1}\;+\;\beta_{2}x_{2}\;+\beta_{3}x_{1}x_{2} \end{array}$$

where *x*_1_ and *x*_2_ each correspond to the at-risk genetic variants, *x*_1_*x*_2_ accounts for the interaction between them, and β_*i*_ are parameters being estimated from the data.

Odds ratios are highly related to logistic regression models. Indeed, exp(β_*x*_) is an estimate of the odds ratio between the outcome and predictor variable *x* when values of other predictor variables are fixed. This is interesting because interpretation of odds ratios is intuitive. An odds is a measure related to probabilities. If an event has some non-null probability to occur in a particular experiment, odds for this event can be viewed has the ratio of the number of events to the number of non-events if the experiment were repeated multiple times. Thus, high odds correspond to high probability for this event, and *vice versa*. Given a probability *p* of occurrence for this event, an odds is defined as follows: $Odds=\;\frac{proportion\;of\;success}{proportion\;of\;failure}=\frac{p}{1\;-\;p}$.

An odds ratio (OR) is then simply the ratio of two odds. It evaluates association between disease occurrence and predictor variables. As such, this measure is closely related to statistical independence: if two variables (in the example below, SNP genotype and disease status) are statistically independent, their OR reduces to 1. Note that an OR not equal to 1 does not necessarily imply a statistically significant association.

**Table 1:** **Example of 2 × 3 frequency table to compute an allelic odds ratio**.

		**SNP genotype**		
		**AA**	**Aa**	**aa**
**Disease status**	**Affected**	a	b	c
	**Unaffected**	d	e	f

Based on Table 1 above, the odds ratio might be calculated using $OR=\;\frac{\left(2\;*\;a\;+\;b\right)∕\left(2\;*\;d\;+\;e\right)}{\left(2\;*\;c\;+\;b\right)∕\left(2\;*\;f\;+\;e\right)}$, assuming allele A is the at-risk allele. This OR is also called the allelic odds ratio (Sasieni, 1997).

Thus, epistasis detection has become an important field of research in human genetics: more complex models are studied nowadays, where combinations of genetic variants are examined for association with a trait. From a biological point of view, it seems unlikely that some phenotypes are only driven by genetic variants acting independently. For instance, large and complex networks of gene-gene and protein-protein interactions are well known in systems biology for their high connectivity, density and resistance to variation (Boone et al., 2007). Moreover, it has been observed that consequences of induced mutations are greatly variable in different genetic backgrounds (Mackay, 2014). Once aware of all this, it seems inconsistent to see gene-gene interactions as rare events.

### Biological epistasis and statistical epistasis

First, it is essential to distinguish biological epistasis (also called functional epistasis) from statistical epistasis (Cordell, 2002). The term biological epistasis was coined by Bateson (1909). In its original definition, it only involved allele effect at one locus concealed by the effect of another allele at a second locus. This can be seen as a broadening of the dominance concept at an inter-loci level. A more recent definition also allows genetic variant effects to be enhanced by effects of other genetic variants (Siemiatycki and Thomas, 1981). Generally, speaking, an epistatic effect exists when the effect of an allele at a genetic variant depends either on the presence or absence of another genetic variant.

On the other hand, statistical epistasis refers to the departure from additive effects of genetic variants at different loci with regard to their global contribution to the phenotype (Wang et al., 2010a). This definition was proposed by Fisher (1918). One relies on this definition when one wants to detect epistatic interactions with computational methods. Ultimately, the goal consists in interpreting interactions found to be statistically relevant in order to get closer to their biological definition and to apprehend the underlying functional mechanisms. This last step is undoubtedly the more difficult one (Moore and Williams, 2005) and is often disregarded.

**Figure 2:**
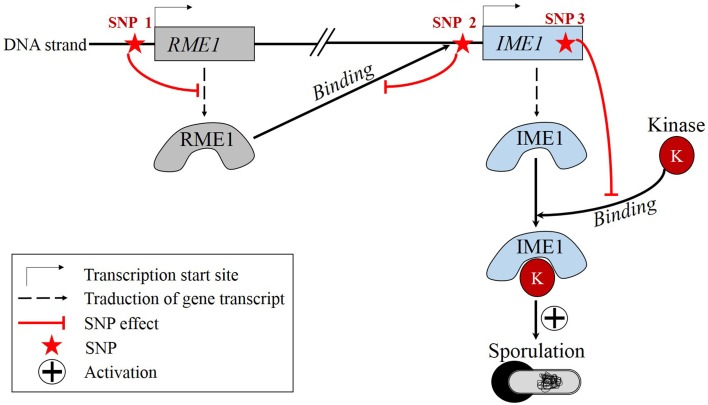
**Real example of epistasis: *S. cerevisiae* sporulation is regulated by epistatic effects among three SNPs**. State of SNP 1 modulates the production rate of RME1. State of SNP 2 influences the binding specificity of RME1. State of SNP 3 conditions the binding specificity of IME1-kinase.

A recent concrete example of epistasis has been described by Gertz et al. (2010), where three SNPs were shown to be involved in an epistatic interaction in yeast *Saccharomyces cerevisiae* (Figure 2). In the following, italic characters refer to the gene while normal characters refer to the corresponding protein. One SNP is located in the promoter region of *RME1* which encodes a transcription factor repressing the transcription of *IME1*, a gene coding for a transcription factor which promotes sporulation. State of this SNP influences the production rate of RME1. The second SNP is located in the promoter region of *IME1*. Its state affects the binding specificity of RME1-*IME1*. The third SNP lies in the coding region of *IME1* and its state conditions the binding specificity of IME1-kinase, which is the active form of IME1. Gertz and coworkers showed that the allele combination of these SNPs have a non-additive effect on the RME1-*IME1* binding and on the sporulation efficiency. Consequently, sporulation efficiency is partly ruled by epistasis. Many other cases of epistasis have been evidenced recently (Smith et al., 2014; Ellis et al., 2015; Huang et al., 2015; Liu et al., 2015; Matsubara et al., 2015).

### Origin of epistasis: an evolutionary point of view

Canalization is a theory proposed by Waddington (1942). It is based on a generally admitted assumption: natural selection maintains the majority of a population into a healthy condition. Thus, in response to genetic and environmental variations, phenotypic modifications are buffered. This is especially true for vital physiological levels, such as blood glucose or blood pressure. To this end, evolution has favored complex robust systems resistant to variations (Moore and Williams, 2009). A compelling argument in favor of this hypothesis is the redundancy rate in biological networks. This feature is well known in systems biology where protein-protein interaction and gene-gene interaction networks exhibit redundant pathways making them resistant to variations (e.g., to deletion of a network node). A disease state would then be due to accumulation of mutations in the genetic network such that its robustness is outstripped. Therefore, all these network interactions are likely to involve epistatic effects. Canalization theory thus explains why so many variants only provide small contributions to the phenotype (Moore, 2003).

### Challenges in epistasis detection

Challenges in epistasis detection are threefold. The first one is statistical. Statistical methods traditionally used in univariate SNP-phenotype associations are not adequate to find epistasis. Finding epistatic interactions is a typical case of the *large p, small n* problem (Johnstone and Titterington, 2009). In practice, the aim is to balance the false-positive rate—produced by the astronomic number of tests performed—and the false-negative rate—a consequence of applying too much stringent significance thresholds. Moreover, SNPs involved in epistatic interactions may have very low minor allele frequencies (MAFs) whereas the number of variants to be tested might be huge. As a result, data is often sparse, leading to the so-called *curse of dimensionality*. The second challenge is computational. Though the overall complexity is linear with the number of individuals in the studied population, it becomes exponential when the interaction order increases. In 2-way interactions, this complexity corresponds to quadratic complexity. The number of combinations to be tested within a dataset containing 1 million SNPs is tremendous: 5 × 10^11^ pairwise interactions, 1.7 × 10^17^ 3-way interactions, 4.2 × 10^22^ 4-way interactions, 8.3 × 10^27^ 5-way interactions, and so on (Ritchie, 2015). Hence, an exhaustive search of epistatic interactions of order 3 or more would lead to a computational burden too prohibitive. Finally, the third challenge is the interpretation of the analytical results. To interpret statistical results biologically is not straightforward, for statistical interaction does not automatically entails interaction at the biological or mechanistic level (Cordell, 2002).

## Exhaustive search for epistasis

In this section, we will discuss strategies of detection that exhaustively test all combinations of variants. Exhaustive search has been proposed to circumvent the local optimality problem, a drawback of heuristic techniques. Most exhaustive methods are designed to detect only pairwise interactions and those directed at higher order detection are simply not scalable. Despite their shortcomings, traditional parametric regression methods serve as a foundation in the field, as emphasized in the following subsection. Then, we will present a strategy derived from such regression methods and designed to be faster than traditional methods. Finally, we will discuss two model-free approaches.

### Parametric regression methods

Traditionally, the most common framework for exploring GWAS data is parametric regression models. A parametric algorithm has a fixed number of parameters that has to be estimated from the data, and relies on strong assumptions about the probability distribution generating the data. This class of algorithms makes accurate predictions when those assumptions are sufficiently close to reality, but performs badly when proved incorrect. Logistic regression (see Box 1) has been widely used as a parametric method for exhaustive search of interactions in association analysis. For example, software PLINK (Purcell et al., 2007) has implemented logistic regression models to detect epistasis. But, in high dimensional data, parameter estimation is a costly and non-accurate procedure that introduces large standard errors because sample sizes are too small compared to genome-wide data size. As a consequence, many false positives are generated when dealing with such data. To overcome this problem, *p*-values are usually corrected with Bonferroni multiple-test correction (see Box 2). This correction being overly conservative, only interactions with very strong effects will be detected and many other interactions will be missed. Hence, the logistic regression strategy has been widely portrayed as unsuitable for handling genome-wide datasets (Cordell, 2009; Moore and Williams, 2009; Steen, 2012). Highly related to standard regression methods, penalized regression techniques, such as the LASSO (least absolute shrinkage and selection operator) or SCAD (smoothly clipped absolute deviation) gained some popularity to detect SNP-SNP interactions. However, those techniques are restricted to two-way interactions and are still prone to inflated false positive rate. Moreover, they are too computationally intensive to exhaustively search through all the pairwise interaction search space. In that case, feature selection techniques are required (further discussed in Section Two-stage Approach: Filters to Obtain Reduced Search Space). The interested reader is referred to Gou et al. (2014) for a recent detailed application of penalized regression-based approach for epistasis detection.

Box 2 **Bonferroni correction.**

*Problem* - Hypothesis-based statistical tests (e.g., *t*-test) are subject to false positive inflation when multiple tests are performed. For example, at a traditional 5% threshold set for statistical significance, there is a 5% chance to falsely reject the null hypothesis. Hence, if this test is performed 100 times when the null hypothesis is in fact true, and 5 tests are found to be statistically significant, then all 5 represent false positive associations. In this case, it is said that the risk is high and uncontrolled. This issue is known as the *problem of multiple tests*.

*Answer* - Bonferroni correction is applied to properly adjust the type I error rate. It consists in dividing the significance threshold by the total number of tests performed. For instance, if a study involves testing for 100 000 hypotheses at a desired global 5%significance level, the corrected significance level for each test is set at $\frac{0.05}{100\;000}={5\times 10}^{-7}$.

*Shortcoming* - This method tends to reject non-null hypotheses due to its conservativeness. This conservative feature is also a shortcoming. It becomes inaccurate because it only favors strongly significant associations. As a result, many true positive associations will be missed (i.e., creating false negatives), thereby leading to a loss in statistical power.

### Bitwise representation of data and likelihood ratio-based testing

**Figure 3:**
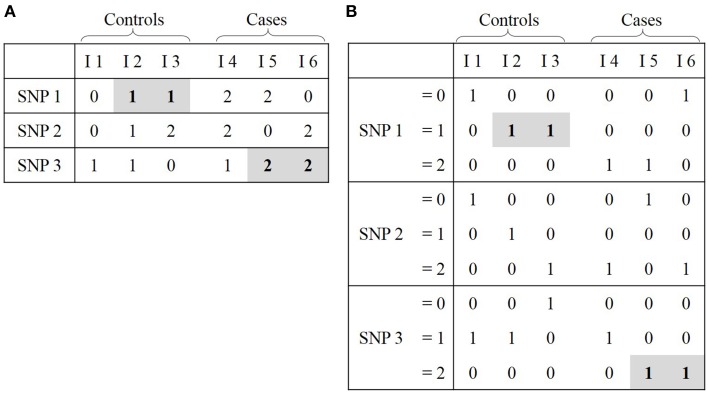
**Representations of GWAS data**. **(A)** Classical representation: cell (i, j) corresponds to status of SNP i for individual j. **(B)** Binary representation: cell (i, j) corresponds to the true (1) or false (0) assertion that a SNP i has a specific value (0, 1, or 2) for individual j. For ease of comprehension, the link between these two representions is highlighted in gray.

We will introduce the Boolean operation-based testing and screening (BOOST) software program to exemplify this section. Designed to be fast, BOOST runs an exhaustive analysis of all potential pairwise SNP-SNP interactions (Wan et al., 2010). The main feature of BOOST is to build contingency tables and use them to calculate log-likelihood ratios for evaluating interaction effects. For two SNPs, a contingency table is a 3 × 3 matrix displaying the frequency distribution of all nine possible genotypes (Figure 1B). However, computing all potential contingency tables at a genome-wide scale is a time-consuming process. In fact, there are as many contingency tables as there are pairwise interactions to test (see Section Challenges in Epistasis Detection). In order to boost the procedure in terms of time and space efficiency, GWAS data is first transformed in a binary way. In usual data representation, each row symbolizes a SNP and each column symbolizes a subject (Figure 3A). In binary representation, each SNP is depicted by three rows, each of them describing the genotype status (i.e., 0, 1, or 2), and two columns depict cases and controls subjects respectively (Figure 3B). Each table cell contains a bit string where each bit represents one subject and its genotype: 1 if it corresponds to the genotype status encoded by the current row, 0 otherwise. Even if the binary matrix seems three times larger than the usual one, its space usage is smaller because one bit is an eighth of a byte, and bytes are the usual units (i.e., non binary) used for storing information. That representation also sticks closer to machine-language, which means that building a contingency table from it only involves fast bitwise (i.e., Boolean) operations.

Once contingency tables are constructed, the program is ready to test for pairwise interactions. The way to detect epistasis complies with Fisher's epistasis definition (see Section Biological Epistasis and Statistical Epistasis) since authors look for a difference between the independent effect model (i.e., marginal effects) and the model which includes both marginal and interaction effects. In other words, for each SNP pair, BOOST tests for a departure from the linear additive model. Under the assumption of equivalence between a logistic regression model and its corresponding log-linear model (Agresti, 2002), this departure is expressed in terms of log-likelihoods. However, the traditional log-likelihood of marginal effect model is constructed via computationally costly iterations that are not tractable at a genome-wide scale. Hence, authors use a non-iterative approximation of the log-likelihood ratio called Kirkwood superposition approximation (KSA) (Matsuda, 2000). On the basis of contingency tables, all pairwise interactions are tested with this indulgent KSA. As it is an approximation, too many false positives are deemed significant with respect to a threshold specified by the user. Therefore, after this first quick screening phase, interaction effects of the selected SNP pairs are again evaluated in a second phase. The number of SNP pairs is supposed to be reduced enough during the first phase in such a way that evaluation of interaction effects via a classical log-likelihood ratio on the remaining pairs is now affordable. Finally, significance of evaluated effects is assessed with a χ^2^ test. One could say that the use of the χ^2^ statistic discredits the method with the following argument: testing interaction effects of a SNP that shows high marginal effect with a χ^2^ statistics may lead to evidence of a statistically significant epistatic effect while that perceived signal could solely be due to noise induced by high marginal effect. For instance, the latter issue has been reported in 2013 by Goudey and coworkers in their result section (Goudey et al., 2013). As a consequence, this phenomenon could favor the selection of many false positive interactions that have little to no epistatic effect. However, even if BOOST uses the χ^2^ statistics to ultimately assess significance of epistatic interactions, tested SNP pairs already show significant association with a log-likelihood difference between the model which does not consider interactions (reduced model) and the model that does consider them (full model).

This approach is faster than its contemporary Bayesian method BEAM (see Section Bayesian Networks) and shows comparative power of detection. A year later, an even faster version that relies on graphic processing units (GPU) instead of central processing units (CPU) was developed. However, an important shortcoming arises because BOOST heavily relies on contingency table construction: low minor allele frequencies (MAF) generate sparse contingency tables, which hampers the detection power of BOOST. Indeed, in each cell of the contingency table, a minimal number of individuals is required so that the χ^2^ test is statistically valid. But when contingency tables are sparse, this requirement is not met, thus leading to failure of epistatic interactions detection. Despite the fact that nearly all true positives are detected (i.e., the detection power is high), BOOST is sensitive to type I errors (Yoshida and Koike, 2011). Finally, a notable shortcoming is that the method only analyzes pairwise interactions and no higher order interactions.

### ROC curve analysis

Goudey et al. introduced the genome-wide interaction search (GWIS) model-free approach in 2013 with the purpose of pairwise epistasis detection (Goudey et al., 2013). While BOOST compares a difference in segregation between two regression models, GWIS tests the difference in segregation power between a SNP pair and the corresponding SNPs taken individually. GWIS is not based on regression analysis, but exploits receiver operating characteristic (ROC) curves to test the discrimination power of SNP pairs. A ROC curve plots the true positive rate (i.e., sensitivity) against the false positive rate (i.e., 1 – specificity) of a classification model. In the context of GWAS, a ROC curve represents the performance of some model designed in classifying individuals according to their affected or unaffected status. For each pair of SNPs, GWIS considers three classification models and builds the respective ROC curves: two for each SNP taken individually, and one for the SNP pair. When the ROC curve corresponding to a SNP pair lies over the other two curves corresponding to individual SNPs, the SNP pair is said to have better prediction power than SNPs taken individually. The next question is to assess if the departure in prediction power between these classification models is significant. To answer this question, Goudey et al. proposed a model-free hypothesis test called *difference in sensitivity and specificity* (DSS). The goal is to quantify the gain in sensitivity and specificity of a ROC curve over another one (Goudey et al., 2013). It seems important to the authors to perform exhaustive search rather than heuristics, in order to avoid being trapped in local optima, then missing significant pairs. GWIS is also designed to be fast (e.g., faster than BOOST) and to scale up to datasets containing millions of SNPs.

The BOOST and GWIS strategies are designed to run exhaustive genome-wide fast scans of epistatic interactions. However, they are restricted to the detection of interacting SNP pairs, which is a substantial limitation. All epistatic models assuming interaction with order greater than two will be missed by these two methods. In the next section, we present a technique that overcomes this problem and exhaustively looks for higher order epistasis.

### A full combinatorial approach

Multifactor Dimensionality Reduction (MDR) is now a reference in the epistasis detection field. No parameters are estimated (i.e., nonparametric) and no assumptions are made on the genetic model (i.e., model-free) under this supervised classification approach. This strategy could detect interactions even when independent main effects are inexistent (Ritchie et al., 2001, 2003; Hahn et al., 2003). It is not constrained to identification of pairwise interactions but also searches for higher order interactions (Moore et al., 2006).

**Figure 4:**
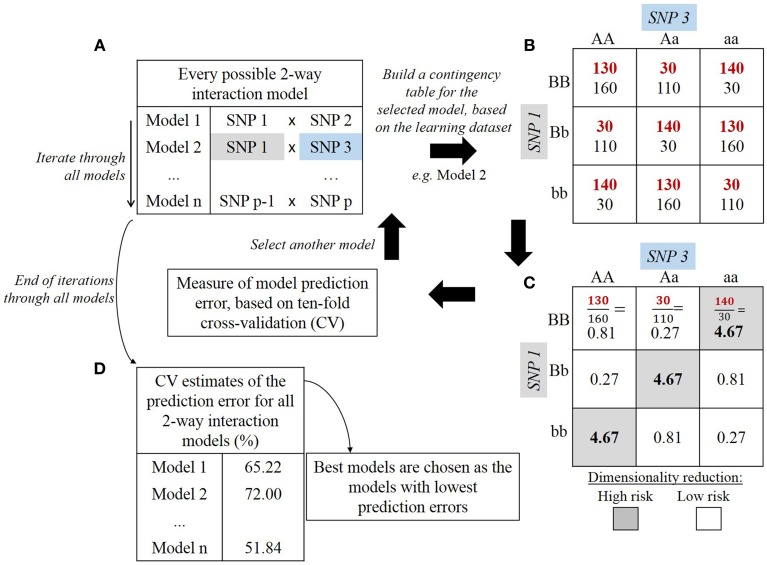
**Steps of multifactor dimensionality reduction (MDR) algorithm: example of 2-way interaction model**. Description of one iteration of the cross-validation process. In **(A)**, a SNP combination is drawn among all potential SNP combinations. In **(B)**, numbers in red denote counts for cases whereas numbers in black denote counts for controls. In **(C)**, each cell displays the ratio of cases over controls. In **(D)**, the prediction error is estimated over the 10 iterations.

First, MDR partitions the dataset for cross-validation. By default, nine tenths of the dataset (training set) is used to build the model and the remaining tenth (testing set) is used to evaluate this model. The model is built following the steps presented in Figure 4. For an interaction order specified by the user, the corresponding number of SNPs is drawn (Figure 4A). Genotype combination counts are then distributed into a contingency table (Figure 4B). For instance, in a two-SNP biallelic interaction model, the nine possible two-locus genotype combinations are allotted into their respective table cells. For a three-SNP interaction model, twenty-seven table cells would be needed. Then, the count of cases and controls is reported for each genotype combination and each cell is evaluated with the following ratio: $\frac{number\;of\;cases\;sharing\;this\;genotype\;combination}{number\;of\;controls\;sharing\;this\;genotype\;combination}$ (Figure 4C). This way, each genotype combination is classified either as high-risk if the above ratio lies beyond a specified threshold (e.g., 1.0), or as low-risk if it lies below that threshold (De et al., 2014). The classification model is then formed by merging cells marked high-risk in one group and all cells marked low-risk in another group. This explains why that method refers to “Dimensionality Reduction”: starting with a problem where dimensionality equals the chosen interaction order, only one dimension remains in the end with high-risk and low-risk values. These steps are repeated for every possible combination of SNPs at a given interaction order, and each combination results in one prediction model. A 10-fold cross-validation process allows to assess the quality of such models. In other words, for each of the 10 iterations of the cross-validation, the models are trained to discriminate between low-risk and high-risk groups through the learning step (on nine tenths of the data). The proportion of ill-classified affected and unaffected individuals is evaluated on the testing set (one tenth of the data). Finally, the prediction error of each model is estimated over the 10 iterations (Figure 4D). The top best models over the 10-fold cross-validation are retained.

As the main feature of MDR is to reduce the data dimension, it can easily be combined with other classification methods (Moore and Andrews, 2015). This flexibility is also a good point to emphasize because since 2006, many extensions of MDR have been proposed so that it is applicable to quantitative traits (Gui et al., 2013). Besides, other variants of the MDR algorithm have been proposed that rely on parallel implementations to boost MDR computing time performance (Bush et al., 2006), to handle missing data (Namkung et al., 2009), or to implement permutation tests (Greene et al., 2009a). However, MDR remains a brute-force search algorithm that induces a prohibitive computational burden when the number of SNPs to analyze exceeds several hundreds. This lack of scalability is its most critical shortcoming in a genome-wide analysis context.

Most exhaustive strategies cannot afford screens of higher order interaction space search since they are not designed to scale up (Taylor and Ehrenreich, 2015). Even the aforementioned GWIS method is restricted to pairwise interaction detection. Exhaustive methods allowing exploration of higher order interactions, like MDR, cannot handle a genome-wide analysis and are constrained to several hundreds of SNPs. To overcome this shortcoming, a common technique is to preprocess data, reducing the entire SNP set to a smaller subgroup that has a tractable size for exhaustive higher order genetic interaction analysis. However, the type of filter is also important. Choosing a marginal-effect dependent filter would be indeed counterproductive with a method like MDR which is most effective in detecting interactions showing pure epistatic effects.

## Two-stage approach: filters to obtain reduced search space

To address the computational burden issue, the overarching goal of some methods is to restrict the analysis to a small subset of candidate markers so that the exhaustive investigation of the remaining combinations is computationally tractable, even for higher order interactions. One approach is to conduct a single SNP-SNP analysis to keep only SNPs with significant marginal effects. SNP combinations are then tested among the remaining marker subset. For example, this strategy has been used in combination with stepwise logistic regression to pre-select a small fraction of SNPs (e.g., pre-determined 10%) based on single-SNP associations significance, before testing for interactions between the selected markers (Marchini et al., 2005). But such filtering leads to an obvious bias where epistatic interactions exclusively induced by combinatorial effects (i.e., with no marginal effect) are not picked up. Nevertheless, there are other ways to reduce the number of SNP combinations down to an informative subgroup. There also exists data mining and data integration techniques dedicated to filter and score downsized genetic variant sets, where null marginal effect is not a rejection condition. We will illustrate each technique in the next two subsections.

### Filtering based on data mining techniques

We will illustrate this category with the ReliefF method. ReliefF approach consists in learning informative features from the dataset without any *a priori* knowledge (Robnik-Šikonja and Kononenko, 2003). The algorithm computes a proximity measure between individuals on the basis of genome-wide genetic similarity. The goal is to evaluate the quality of genetic variants according to how well their values distinguish individuals near to each other.

**Figure 5:**
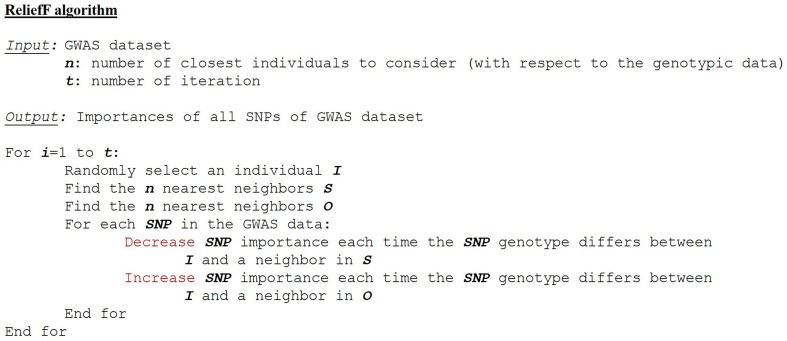
**ReliefF algorithm**.

The algorithm is quite simple (Figure 5). For each individual (noted *I*), the procedure determines the nearest individuals (i.e., neighbors) sharing the same phenotype (set noted *S* for *same*), and also the nearest individuals that show up the opposite phenotype (set noted *O* for *opposite*). If *I* and *S* show different values for a marker, then this variant discriminates individuals having the same phenotype, thus decreasing its importance. On the contrary, if *I* and *O* show different values for a marker, this variant discriminates individuals having different phenotypes, thereby its importance is increased. These steps are then repeated over a predefined number of individuals. Moore and coworkers showed in 2007 that ReliefF algorithm is scalable (Moore and White, 2007).

The popularity of ReliefF gave rise to several variations (Kononenko, 1994) that we will quickly present below. RReliefF (Regressional ReliefF) was designed to study quantitative traits like eQTL epistasis (Huang et al., 2013). When applied to a genome-wide dataset, noisy genetic markers may be attributed too much weight, hence inflating their importance estimates. To alleviate this problem, TuRF (Tuned ReliefF) proposed to eliminate from the SNPs set considered for epistasis detection, SNPs with no or very low importance. These SNPs rarely discriminate individuals from their neighbors having a different phenotype (Moore and White, 2007). Importance of remaining SNPs is then re-estimated, without considering these noisy SNPs. Results are encouraging since TuRF power of detection is identical to or better than ReliefF. ECRF (Evaporate Cooling ReliefF) also attempts to solve the noisy variable problem (McKinney et al, 2007). It significantly outperforms ReliefF for detecting epistasis. Its algorithm combines information theory and ReliefF. In ReliefF and its above extensions, the user-defined number of nearest individuals to consider (i.e., *S* and *O*) is usually fixed at 10. Using such a predefined number may be considered as a selection bias since the information coded in the data is not fully exploited. To tackle this issue, SURF (Spatially Uniform ReliefF) proposes to take into account all neighbors within a given distance rather than a fixed number of neighbors (Greene et al., 2009b). SURF generally takes into consideration much more neighbors than ReliefF, labeling 25–50% of all individuals as neighbors. So when applied to a GWAS dataset, SURF has higher power of detection than ReliefF, albeit this may become a cumbersome procedure. A latest variation, SURF* (Greene et al., 2010), also considers information of farthest individuals to build importance scores. In terms of detection power of epistatic interactions, the performance of TuRF and ReliefF has been compared in Moore and White (2007). ECRF has also been compared to ReliefF in McKinney et al (2007). Finally, SURF has been compared to both ReliefF and TuRF in Greene et al. (2009b). However, ECRF and SURF have not been compared to each other, as well as ECRF and TuRF. ECRF and TuRF show improved performance over ReliefF, whereas SURF and SURF^*^ show improved performance over both ReliefF and TuRF.

### Filtering based on data-integration techniques

Another research area advocates the use of knowledge from external databases, in order to select SNP groups that are relevant to the phenotype of interest (Grady et al., 2011). Even if this approach is hindered by a lack of epistasis understanding in complex organisms, it avoids the black box effect of data mining techniques that may hamper the interpretation of underlying biological mechanisms.

One way to do that is to query information in online public protein-protein interaction databases like IntAct (Kerrien et al., 2012), BioGRID (Chatr-Aryamontri et al., 2015), STRING (Franceschini et al., 2013) or ChEMBL (Willighagen et al., 2013). It is then possible to narrow all SNPs down to a reduced list of markers located in genes that encode for proteins involved in relevant interactions. When markers are mapped to an interacting gene pair, tests are exhaustively conducted on interactions between each SNP of the first gene against each SNP of the second gene. Unfortunately, one would probably fail at discovering new biological models by selecting SNPs in such a direct way. A more promising strategy is to come up with a score for each SNP (Ritchie, 2015), based on assessed relative importance of the proteins encoded by the genomic region encompassing the SNP. Novel findings are within reach by running a prioritization scheme rather than a strict removal (Pattin and Moore, 2008).

Resorting to pathways is also interesting. For instance, this approach has already been applied with information drawn from pathways involved in lipid synthesis (Ma et al., 2015), by including evidence from public databases like KEGG Pathway (Kanehisa et al., 2012), Reactome (Croft et al., 2014) or BioCarta (Nishimura, 2001). For a pathway of interest, one first looks at the involved genes, and then maps SNPs to these genes. The technique is similar to the above protein interaction-guided analysis. But there is a bias as certain pathways are more deeply studied than others: genes (and SNPs therein) involved in a very well-known pathway may be given more weight than those involved in a less studied one. Instead of relying on guidance restricted to pathways or to protein-protein interactions, the comprehensive knowledge approach (Pendergrass et al., 2013a) is more global as it exploits pathways, protein interactions, gene expression, gene ontology, etc. As appealing as this approach might be, it is not currently possible to accurately evaluate results found by this strategy because implementing pathway simulations is not a trivial task. This would require a tool designed to simulate pathways and protein-protein interaction networks, and then simulate GWAS data where several SNPs are involved in these networks. Such a tool does not exist yet. Therefore, for this kind of filter based on comprehensive knowledge, we cannot properly and objectively assess its scientific relevance.

One software program worth mentioning is Biofilter. It gathers information from 13 databases (Pendergrass et al., 2013a), which contain experimental evidence of interaction, pathway or ontological similarity relationships. On the basis of biological plausibility, Biofilter models interactions that will be tested irrespective of the marginal effects. So it creates polygenic models, thanks to gene-disease and gene-gene connection knowledge (Pendergrass et al., 2013b). The statistical and computational challenges are also addressed since not all combinations of interactions are examined. Statistical relevance is based on the statement that the more two genes are involved in a relationship, the more likely they are to share an important biological link (Bush et al., 2009).

Although data-integration techniques yield meaningful and biologically relevant results, exploiting external information sources like pathways or protein-protein interaction networks is controversial. Online databases are incomplete and so is our understanding of biological pathways. Thus, making use of them to build filters would in most cases results in a flawed analysis. Moore and Hill recently recommended (Moore and Hill, 2015) to combine both the *biased* approach (from a biologist point of view) based on expert knowledge, and computational approaches solely driven by GWAS data (neither immune to bias from a statistician point of view). Similarly to computational exhaustive methods, this combined approach is taking advantage of artificial intelligence methods, which we discuss in the next section.

## Non-exhaustive searches enhanced by artificial intelligence

Machine learning and combinatorial optimization represent alternatives to parametric statistical methods for detecting combinations of variants that are associated with a phenotype. Machine learning methods build non-parametric models to compile information further used for epistatic detection. Combinatorial optimization techniques consider a search space of solutions (i.e., combinations of potentially interacting SNPs) and browse through this space to find the more relevant combinations. Heuristics are commonly used in these algorithms, especially when dealing with genome-wide datasets in search of higher order genetic interactions. Identification of classification variables and interactions between them which allows outcome prediction is a well-known hurdle addressed by the machine learning and data mining fields of artificial intelligence (Cordell, 2009). In such non-parametric models, precautions must be taken to avoid overfitting (see Box 3). It has to be noted that if the model complexity of the underlying genetic mechanisms is too high compared to the sample size, using non-parametric methods may not be affordable. In this case, parametric methods are the only practical alternative, assuming that the model assumptions are not severely violated.

Box 3 **Overfitting.**

The aim of machine learning is to explain a system by learning a model with a training dataset. But dataset's particularities result in an overly tuned model adjusted for very specific features (Leinweber, 2007). In other words, overfitting happens when the training stage gives too much importance to the noise within data. Overfitting is detected when a simpler and more accurate model exists. However, identifying what to ignore in the overfitting model is a non-trivial task. Overfitting typically arises when model complexity is too high compared to the size of the training data. In practice, cross-validation possibly combined with pruning is used to avoid overfitting.

A majority of these heuristics test for associations of variants allowing interactions, rather than testing for interactions themselves. The distinction lies in the following: besides SNPs involved in epistatic interactions, a model representing associations allowing for interactions also includes SNPs which have marginal effects. Therefore, although it is not a straight proof of epistasis, it is nonetheless an examination of polygenic models. Thus, if such procedures heavily rely on marginal effects for association findings, they will detect multiple SNPs with independent effect. But if they do not rely on marginal effects, they will also consider epistatic interactions.

With regard to machine learning techniques, we will first take a look at random forests and their variants, then move on to Bayesian network-based strategies. As for combinatorial optimization strategies, ant colony optimization and computational evolution system approaches will be presented.

### Random forests and their variants

A tree-based algorithm generates a tree where each tree-node represents a predictor variable and a path designates a sequence of predictor variables from the root to the leaves of the tree. When the tree is constructed from GWAS data, each node represents a SNP. A basic tree-growing algorithm is deterministic in that each step looks for the predictor variable that optimally segregates the population. So a grown tree is a classifier which represents a SNP set allowing prediction of the phenotype of interest. This approach can handle SNPs that are associated in a non-linear way, dealing with interactions encoded in a hierarchical fashion between layers of the tree. A notable shortcoming of tree-based methods is that they are quite dependent of marginal effects. At the beginning of the tree learning step, the algorithm looks for a single SNP that well discriminates cases from controls. In practice, this is equivalent to looking for SNPs with high marginal effects.

Random forests were designed to avoid bias generated by growing a single tree. The random forest strategy creates multiple—generally thousands—classification or regression trees (e.g., CART) in order to apply an ensemble procedure. An ensemble procedure aggregates the predictions of all trees to produce a powerful and robust prediction tool (Breiman, 2001). The SNP set output is defined as the most important variable set of the random forest (to be further explained in this section). Although growing a random forest is a relatively computationally intensive procedure, it has been evaluated as a good strategy for detecting the most predictive SNPs in large-scale association studies (Bureau et al., 2005) and was applied to GWAS several times in the last 5 years with epiForest (Jiang et al., 2009), random Jungle (Schwarz et al., 2010) and SNPInterforest (Yoshida and Koike, 2011).

**Figure 6:**
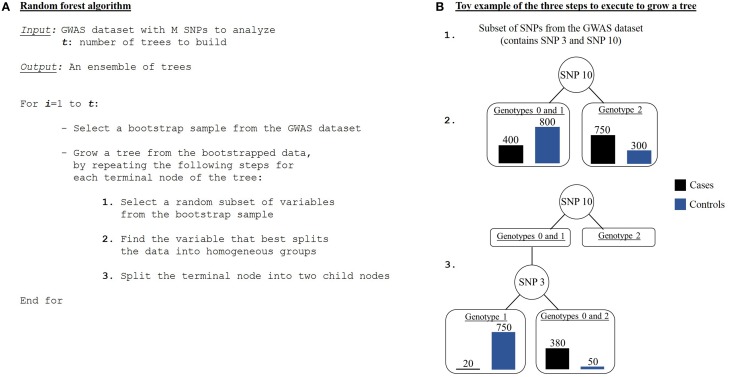
**Random forest algorithm. (A)** Algorithm of a random forest procedure. **(B)** An example of the three steps needed to grow one tree.

A classification tree is grown using the following steps (Jiang et al., 2009). First, a bootstrap sampling is performed from the GWAS dataset comprised of N individuals and M SNPs. It consists in randomly selecting, with replacement, N individuals from the N original individuals. Individuals not drawn are called out-of-the-bag (OOB) individuals. So a new dataset and an OOB set are created for each grown tree. Then a random feature selection is applied to construct each node of the tree. To do so, instead of considering all variables from the initial GWAS dataset, a random subset of variables is picked out without replacement. A recursive data splitting procedure is next executed, such that a parent node results in two child nodes given a rule that leads to a better discrimination of the current set of individuals (from the parent node) with regards to the disease status. This discrimination score is measured as a goodness of split or a decrease in impurityΔ*i*. The tree is then grown up to its largest extent. These previous steps are repeated until a forest is built (Figure 6).

For each node, a so-called variable importance is assessed to evaluate its contribution to the trait either individually or via multi-way interactions with other predictor markers. In other words, variable importance represents weight approximating the causal effect of a predictor variable. There are several ways to measure variable importance (Schwarz et al., 2010). One is the *Gini importance*, a second one is the *permutation importance*, and a third one is the *conditional variable importance*, based on permutation importance. The conditional variable importance seems to be more suitable when applied to genetic data while the other two are biased in presence of linkage disequilibrium (correlation between SNPs) (Strobl et al., 2008). Compared to the original random forest construction, algorithms readjusted for epistasis detection include multiple SNPs at each tree-node during tree building (Botta et al., 2014). It is intended to detect SNP combinations even when marginal effects are very weak or inexistent (Yoshida and Koike, 2011). The readjusted method is less sensitive to SNPs presenting little marginal effects than an exhaustive approach like MDR. However, even if random forests reveal associations potentially pointing at interactions, they cannot make a distinction between a scenario of interacting SNPs and a scenario of several independent SNPs additively contributing to the phenotype. As a result, random forests are lacking clear interpretation.

More recently, another tree assembling software program was developed: GWGGI (Wei and Lu, 2014). It differs from the previous methods in two points. First, it uses a tree-growing algorithm which is more computationally efficient (Lu et al., 2012): the standard variable selection procedure is replaced with a forward algorithm. The principle of a forward algorithm is to take into account previously selected variables. The novel variable identified is the one, when added to the previous set of variables, allowing for the most accurate prediction. Secondly, the GWGGI algorithm relies on likelihood ratios and the Mann-Whitney statistics to assess the predictors' importance in order to facilitate the statistical significance assessment of selected association models. Since each tree can be considered as a multi-locus genotype model, each individual is confronted to each grown tree and a likelihood ratio is generated: $LR_{i}^{t}=\frac{P\left(G_{i}^{t}|D\right)}{P\left(G_{i}^{t}|\bar{D}\right)}$ where $G_{i}^{t}$ is the genotype of individual *i* mapped *t*, and *D* (*resp*.$\bar{D}$) is the control status (*resp.* case status). Then for each individual, all likelihood ratios are assembled into a unique one by averaging the total number of trees. Finally, a *U*-statistic is constructed with comparisons between assembled likelihood ratios of cases vs. controls in order to evaluate the joint association of the selected SNPs with the phenotype (Wei et al., 2013). The *U*-statistic is calculated in the following way: $U=\frac{\overset{N\text{\_}cases}{\underset{i\;=\;1}{\sum }}\overset{N\text{\_}controls}{\underset{j\;=\;1}{\sum }}\psi \left(LR_{i},LR_{j}\right)}{N\text{\_}cases*N\text{\_}controls}$. The ψ function is a kernel function defined as:

$$\begin{array}{l} \psi \left(LR_{i},LR_{j}\right)=\{\begin{array}{c} 1\;if\;LR_{i}\;>\;LR_{j}\\ 0.5\;if\;LR_{i}\;=\;LR_{j}\\ 0\;if\;LR_{i}\;<\;LR_{j} \end{array} \end{array}$$

The null hypothesis states that there is no association between the selected SNPs and the phenotype.

### Bayesian networks

Bayesian networks provide a compact representation of dependencies between variables. A Bayesian network consists of two components: a graphical one and a probabilistic one. In the former—directed acyclic graph (DAG)—variables are represented by nodes and dependencies between them are represented by directed edges. The probabilistic component of a Bayesian network associates a probability distribution with each node of the DAG, thus accounting for uncertainty. A Bayesian network encodes the Markov property: each variable is independent of its non-descendants, given its parents in the DAG. The governing theorem of a Bayesian network is the following. Let *X, Y*, and *Z* be variables of the Bayesian network. If *P*(*X*|*Y, Z*) = *P*(*X*|*Y*), then X is conditionally independent of *Z*, given *Y* (noted *X* ⊥ *Z*|*Y*). When applied to genetic data, variables are typically SNPs and phenotypic values. Bayesian networks offer an appealing and intuitive way to capture relationships existing between genetic markers and disease status. The structure learning of a Bayesian network amounts to a model selection problem. Because this learning is an NP-hard problem (Chickering et al., 2004), specific techniques have to be used to reduce the computational burden.

A famous Bayesian network-based software program called BEAM (Bayesian Epistasis Association Mapping) (Zhang and Liu, 2007) is often used as a Bayesian-based reference for performance comparisons. BEAM relies on a Markov Chain Monte Carlo (MCMC) algorithm to test iteratively each marker, conditional on the current status of other markers. For each marker, the algorithm outputs its posterior probability of association with disease. Markers are then distributed into three groups: group 0 for markers unlinked with the phenotype, group 1 for SNPs that contribute independently to the phenotype (additive model) and group 2 for SNPs that influence the disease risk given particular allele combinations (epistasis model). After that partitioning phase, a B-statistic is used to further filter detected SNP groups. When the BEAM method was originally published, the B-statistic was a new alternative to the usual χ^2^ test of association between a phenotype and a set of SNPs. A detailed explanation of its computation would require a much deeper presentation of BEAM, which is not the aim of this section. The interested reader is referred to Zhang and Liu (2007) for a comprehensive explanation of how to build a B-statistic. Although the B-statistic enables to get rid of expensive permutation tests, MCMC iterations make this method inadequate when handling datasets containing more than 500,000 genetic markers, which is now commonplace in GWAS studies.

More recently, Han et al. (2012) also worked with Bayesian networks to capture SNP-disease associations with EpiBN. As these authors consider that SNPs are causal with respect to the phenotype, the Bayesian network built here is composed of two layers: one layer with the phenotype as a unique node, connected to parent nodes of the phenotype in the second layer which represents the SNPs associated with the phenotype. Edges between nodes representing SNPs can exist, thus allowing detection of interactions between genetic variants in the model. Instead of a MCMC-based algorithm, they use a Branch-and-Bound iterative procedure to learn the structure of the Bayesian network. At each iteration, the algorithm adds, deletes or reverses an edge. Then a score function is called to find the best network structure evolution since the previous iteration. The network is iteratively constructed and at each iteration, the current network structure goodness is assessed with a score function. The goal is to maximize this score. The score function is made of two terms that indicate how well the current structure fits the data—on the basis of a maximum likelihood ratio—and how complex the Bayesian network is. In Han et al. (2012), it has been shown through multiple simulations that the EpiBN software program seems to outperform BEAM in interactions detection power.

**Figure 7:**
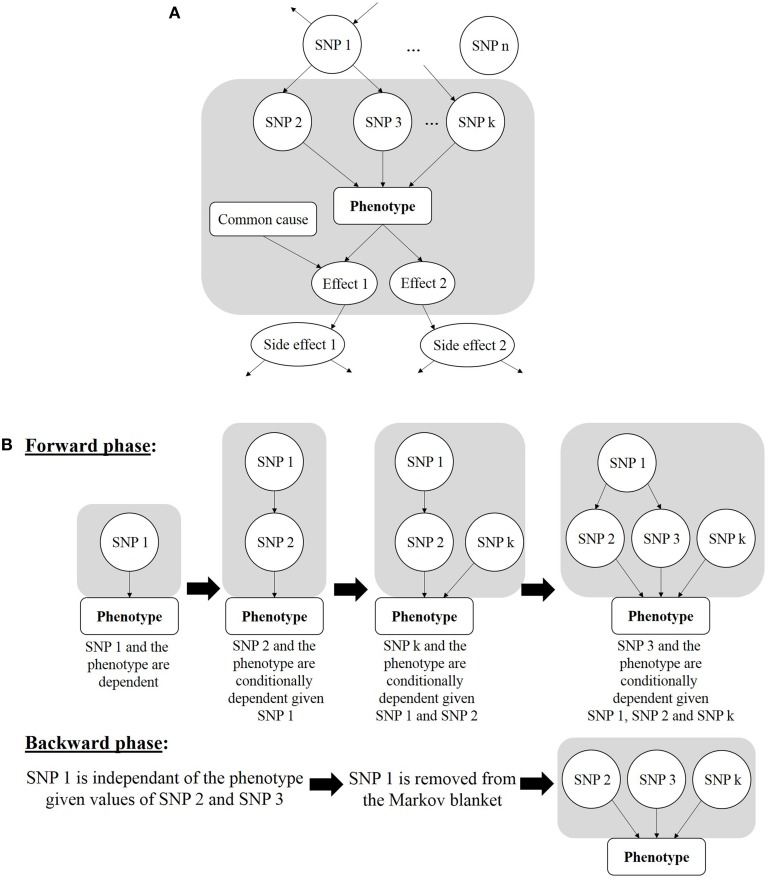
**(A)** Markov blanket of a phenotype, in gray area. It is made of the parents (SNP 2, SNP 3, and SNP k), of the children (Effect 1 and Effect 2) and of the spouses (Common cause of Effect 1 with respect to the Phenotype). **(B)** Two stages of Markov blanket learning. For ease of reading, the Markov blanket is reduced to parents from **(A)**.

A different but not less appealing Bayesian strategy is the Markov blanket-based method. It allows discovery of SNPs in the local pathway of the phenotype, also referred to as “local causal SNPs” (Alekseyenko et al., 2011). In the context of GWAS, this strategy is used to avoid the time-consuming training processes like tree-growing of random forests or structure learning of a full Bayesian network. The principle is to find a minimal set of variables that completely shield the disease status from all other variables, thus resulting in a local Bayesian network fraction that borders the phenotype node in the graph: this set is defined as the Markov blanket. In other words, each SNP will be statistically independent of the case-control status when conditioned on the SNPs forming the Markov Blanket. A Markov blanket-based strategy can be applied for causal findings because the Markov Blanket contains direct causal variables (i.e., parent nodes), direct effect variables (i.e., child nodes), and direct causal variables of direct effect variables (i.e., spouses) (Figure 7A).

With the goal of finding a minimal SNP set, this strategy is expected to minimize the number of false positives. Besides its classification accuracy, this strategy has been put forward for its compactness (Aliferis et al., 2010a). Moreover, the Markov blanket-based strategy has proved to properly address the combinatorial hurdle raised by epistasis analysis at the GWAS scale (Aliferis et al., 2010b). The Markov blanket construction algorithm will generally go through two stages (Figure 7B). The first one, called “forward phase,” adds new relevant variables to the candidate Markov blanket (noted *canMB*). In practice, this stage consists in finding the SNP *X* which is the most associated with the phenotype, given *canMB* (e.g., tested with a G^2^ test, which is a subclass of likelihood-ratio tests and is similar to a χ^2^ test, McDonald, 2014), and including *X* in *canMB* if *X* is dependent of the phenotype, given *canMB* (e.g., if the G^2^ statistics is lower than some user-specified threshold): ¬(*X Phenotype* | *canMB*) ⇒ *add X in canMB*. This operation is repeated until *canMB* no longer changes from one iteration to the other. The second phase, called “backward phase,” aims at removing false positives that were included in the previous step. To achieve it, each SNP of the candidate Markov blanket is checked. A SNP *Y* is detected as a false positive if it is independent of the phenotype given a SNP subset of *canMB*. Three implementations of this approach were recently developed: DASSO-MB (Han et al., 2010), TIE^*^ (Alekseyenko et al., 2011; Statnikov et al., 2013) and IMBED (Yanlan and Jiawei, 2012), and all proved to be more sample-efficient than BEAM, i.e., less samples are needed to reach the same power of detection as BEAM. In DASSO-MB (Han et al., 2010, Han and coworkers postulate that, in epistatic interaction studies, only causal SNPs are sought, and consequently only parent nodes of the phenotype have to be detected. Hence, DASSO-MB represents a more specific application of the Markov Blanket approach. Considering a set of 19 SNPs already known to be associated with rheumatoid arthritis, an application of TIE^*^ (Target Information Equivalency) showed that a Markov blanket-based approach could make the whole SNP set independent of the phenotype when conditioned on three other SNPs identified in the Markov blanket (Alekseyenko et al., 2011). In other words, the reported SNP set does not provide any predictive information about the disease status beyond that brought by the three SNPs identified with the Markov blanket.

The bias of this approach is that the first SNP added to the candidate Markov blanket is picked on the basis of a univariate test. So the detection of marker combinations when marginal effects are slight or nonexistent is still a major obstacle (Han and Chen, 2011). Markov blanket-based strategies also heavily rely on the *faithfulness* assumption, defined with respect to the sample, as follows: every conditional independence in the Bayesian network also exists in the probability distribution of the variables. In practice, this hypothesis is rarely met in GWAS.

### Ant colony optimization

**Figure 8:**
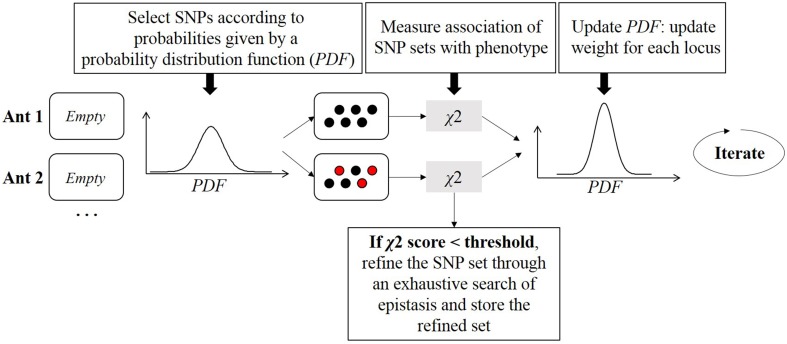
**Ant colony optimization procedure**. For each ant, multiple SNPs are drawn. The probability distribution function (PDF) gives the probability of each SNP to be drawn. Once an ant is filled with a SNP set, joint association of this SNP set with the phenotype is evaluated with a χ^2^ test. For each ant, the PDF is updated according to *p*-values resulting from χ^2^ tests, such that SNPs efficiently classifying individuals will have a higher probability of being drawn in the next iteration.

Ants communicate with each other through pheromone levels to find the optimal path leading to food. If an ant finds a shorter path, it will produce and increase the pheromone concentration along this path. Other ants will more likely follow that path showing increased pheromone concentrations, thereby creating a positive feedback to find the best path to food. In 2010, AntEpiSeeker algorithm (Wang et al., 2010b) was derived from the generic ant colony optimization (Dorigo and Gambardella, 1997) (ACO) algorithm. AntEpiSeeker performs the search of multiple groups of SNPs associated with the disease in parallel. The algorithm is an iterative procedure where artificial ants cooperate at each iteration to update knowledge about the propensity of SNPs to be related to the disease (Figure 8). From a computational point of view, ants represent SNP sets that have potential epistatic effects, and a pheromone concentration is a weight evaluated by epistatic interaction significance of the selected set of SNPs. Communication between ants is mimicked by a probability distribution function (PDF) shared by all ants. The PDF is a function describing the probability of selecting a specific SNP at a specific iteration. This probability depends on the pheromone concentration for this SNP at this iteration, and on another factor which allows to weight SNPs according to expert knowledge drawn from additional biological data. At each iteration, multiple SNPs are picked up, depending on the PDF, to build each ant. Then a χ^2^ test is used as a score function to measure the association between an ant and the phenotype. Results are used to update the PDF for the next iteration. Once highly suspected sets of SNPs are assembled, AntEpiSeeker conducts a second analysis stage: an exhaustive search of epistasis within each built ant is performed, as well as within the set of SNPs that have the highest pheromone levels. The ant colony strategy was also exploited more recently in MACOED (Jing and Shen, 2015).

The positive feedback effect represents an interesting feature of the algorithm. Unfortunately, many parameters require fine tuning, like the number of iterations, the order of interactions, the number of SNPs in each ant, or the evaporation rate of pheromones which is an ingredient of the update function of the PDF. Those parameters must be estimated a priori, which is considered as a limitation of this algorithm.

### Computational evolution system

The algorithm behind the Computational Evolution System (CES) is an original strategy based on natural selection and Darwinian evolution. The goal is to grow a computer program from several basic building blocks, similar to a DNA strand emerging from a composition of the four basic nucleotides. This program tries to reproduce the natural evolution process underlying complex real biological systems. The first question is what the building blocks are, whenever one wants to build such a computer program. The answer is non-trivial and is decisive in epistasis analysis when trying to avoid dependence to marginal effects. In a recent application of CES (Moore and Hill, 2015), the building blocks were defined as basic functions involving SNPs. A basic function is an operator (add, delete, and copy) aggregating SNPs in combinations, and the resulting composition of building blocks is called a solution. In other words, a solution can be perceived as a set composed of various elements, where each element is a function dealing with genetic polymorphisms. A solution is thus a classifier designed to predict the case-control status of an individual given its genotype.

A CES is governed by a pyramidal architecture where each level is probabilistically controlled by its upper layer. The lowest level is a two-dimensional grid of solutions where each solution is a list of building blocks. The second level is a grid of solution operators influencing the lower layer. Each cell consists of a combination of add, delete, and copy operators having a given probability of being executed. Attributes can be added, deleted or copied either randomly or using expert knowledge. A third level of computation is used to introduce changes in execution probabilities of the latter operators. A last level controls the variation rate of the third layer. Uncertainty is injected in this architecture in order to mimic a realistic natural evolution system. As a result, there is high flexibility in model creation based on CES.

The stage during which all solutions are modified is called a generation. From one generation to the next, accuracy of each solution is modified as follows: an operator is drawn according to the execution probability distribution; this operator is then applied to each solution. It has to be noted that the initialization of the CES grid of solutions is either random or guided with expert knowledge. This last option has been highly recommended (Greene et al., 2009a; Payne et al., 2010). The accuracy of each solution is assessed in the following way. Each solution is applied to case and control individuals to obtain two distinct score distributions: one for cases and one for controls. A threshold is determined as the arithmetic mean between the medians of the two distributions. Then individuals are predicted to be cases or controls given this threshold. The solution accuracy is computed afterwards as an error ratio between predicted and actual status. Once one knows how to compare solutions, one can select the optimal solution which maximizes the prediction accuracy. The solution is selected among all generations (e.g., 1000 generations) in the following way. Each solution occupies a lattice position in the two-dimensional grid and competes with its neighborhood composed of eight adjacent solutions. Within this neighborhood, the solution with the highest accuracy is selected to replace the central position of that neighborhood.

This approach is interesting in that it allows modeling of complex interactions with few hypotheses. It also has the capability to use expert knowledge, and is well suited for parallelization. However, the computational complexity of the CES strategy precludes a direct analysis of GWAS data with hundreds of thousands SNPs. Such datasets will require a preprocessing step with filtering methods introduced in Section Two-stage Approach: Filters to Obtain Reduced Search Space.

## Discussion

While an exhaustive epistasis analysis has become a quite straightforward task for SNP pairs, higher order interactions search in an exhaustive way is not conceivable at the moment. In this paper, we reviewed main current strategies for epistasis detection: exhaustive ones based on brute-force approach, filtering ones aiming at reducing genome-wide SNP set size, and different machine learning and combinatorial optimization procedures to find SNP associations yielding the best classification power. Table 2 summarizes categories of methods described in this paper and gives representative software programs illustrating each category. In particular, this table highlights characteristics of the largest GWAS dataset analyzed using each software program. Runtimes are indicated, when available, for sequential and parallel versions of each program, for information about scalability.

**Table 2:** **Summary table of strategies reviewed to detect epistasis along with representative software programs and datasets applications**.

**Strategy**		**Software program**	**Exhaustivity**	**Pairwise-restricted**	**Dataset**	**# SNIPs**	**# Individuals**	**Runtime**		**References**
								**Sequential**	**Parallel**	
Bitwise operations and Likelihood ratio tests		BOOST	Yes	Yes	WTCCC–multiple diseases	459,019	5000	NA	23 h (4 CPUs)	Wan et al., 2010
ROC curve analysis		GWIS	Yes	Yes	WTCCC–multiple diseases	459,019	5000	60 h	10.9 h (4 CPUs)	Goudey et al., 2013
Combinatorial		MDR	Yes	No	Simulated	50	500	36 min	NA	Moore et al., 2006
Random forest		Random jungle	No	No	Crohn's Disease	275,153	1003	12.7 h	0.53 h (40 CPUs)	Strobl et al., 2008
		Snplnterforest	No	No	WTCCC - RA	10,000	3500	98 h	NA	Yoshida and Koike, 2011
		GWGGI–TAMW	No	No	WTCCC - CAD	459,019	4864	10 h	NA	Wei and Lu, 2014
		GWGGI–LRMW	No	No	WTCCC - CAD	459,019	4864	3.5 h	NA	Wei and Lu, 2014
Bayesian		BEAM	No	No	AMD	47,727	3500	8 days	NA	Zhang and Liu, 2007
		epiBN	No	No	AMD	96,933	146	NA	NA	Han et al., 2012
	Markov blanket	Dasso-MB	No	No	AMD	91 495	14G	NA	NA	Han et al., 2010
		FEPI-MB	No	No	Simulated	500	4000	0.5 s	NA	Han et al., 2011
		IMBED	No	No	AMD	96,933	146	NA	NA	Yanlan and Jiawei, 2012
		TIE^*^	No	No	NARAC	490,073	2 044	NA	NA	Statnikov et al., 2013
Ant colony optimization		AntEpiSeeker	No	No	WTCCC – RA	332,831	3503	NA	5 days (2 CPUs)	Wang et al., 2010b
Computational evolution system		CES	No	No	Prostate cancer	219	2286	NA	NA	Moore and Hill, 2015

Runtimes were not always available (NA) and are indicated for simulated datasets when no real data application is available. The notation “WTCCC—multiple diseases” stands for “WTCCC—Bipolar Disorder (BD), Coronary Artery Disease (CAD), Crohn's Disease (CD), Hypertension (HT), Rheumatoid Arthritis (RA), Type 1 Diabetes (T1D), and Type 2 Diabetes (T2D).

Despite efforts for developing novel methods dedicated to epistasis detection, genetic variance of complex traits is weakly explained by detected epistatic interactions. This may be due to low detection power of pure and strict epistatic interactions for many of these methods. Much remains to be done to improve power of detection using model-free searches. For instance, the TURF method (see Section Filtering Based on Data Mining Techniques) which excludes SNPs with low predictive power, prior to performing epistasis detection, could be extended to other strategies like random forests, thereby improving detection of epistasis.

Precision of association measure estimates between epistatic interactions and phenotypes can be enhanced by increasing the $\frac{number\;of\;samples}{number\;of\;SNPs}$ ratio. First, increasing the sample size is a way to improve power of epistasis detection. Federating data from laboratories in the context of meta-analysis is a widespread approach, though subject to biases due to heterogeneity of laboratory practices. Second, reducing the number of SNPs to analyze might improve the statistical power under a given hypothesis. For instance, such a reduction of the search space size is possible thanks to systematic methods, like using significant pairwise interactions as a prior basis for the search of higher order interactions. Regarding data integration approaches, biological expert knowledge based-filters are often proposed to guide epistasis analysis. Being a biased approach, it is recommended to run at the same time a procedure without any *a priori* knowledge (Ritchie, 2015). Although development of epistasis detection methods is growing, many methods are hampered in presence of genetic heterogeneity or incomplete penetrance. Random forest-based techniques have been described to efficiently deal with genetic heterogeneity because data is split in different subsets in early stages of the algorithm (Koo et al., 2013). Besides, some of the existing software programs, like BEAM, will soon become unsuitable to GWAS datasets which will keep growing in size so that several millions of SNPs will be the rule rather than the exception. On the other hand, such a huge number of SNP might increase power of existing strategies tailored to handle massive datasets.

An interesting fact rarely discussed in literature describing the strategies reviewed in this survey is the confusing boundary between epistasis and linkage disequilibrium. Because linkage disequilibrium is by definition a phenomenon involving dependence between genetic variants, its frontier with epistatic interactions may be blurred since the aforementioned software programs are designed to detect SNPs that jointly affect the phenotype. This issue is particularly acute for case-only approaches. For standard case/control studies, if estimation of linkage disequilibrium within controls provides the same result as within cases, then the observed linkage disequilibrium does not originate from epistatic interactions.

Development of simulation models dealing with epistasis is also an active research area (Moore et al., 2015). Even if some authors already use various simulation models to estimate efficiency of their algorithms (Beam et al., 2014), these simulation tools lack the complexity of genetic mechanisms observed in real data. For instance, simulation models used in most software programs introduced in the previous sections only generate pairwise epistatic interactions. As a consequence, strategies dealing with higher order interaction detection are not confronted to simulation scenarios involving those types of interactions. Hopefully, such a gap will certainly be filled in the future.

With regard to evaluating association strength several authors rely on *p*-values to sort the best candidate SNPs. However, *p*-values alone do not allow any straightforward statement about the association strength. A *p*-value only estimates the probability of having observed the value of the test statistic under the null hypothesis (i.e., there is no association between the tested SNP and the phenotype) (du Prel et al., 2009). Odds ratio combined with confidence intervals are also widely used measures in GWAS reports.

The need for scalable and powerful strategy to detect SNP-SNP interactions is clearly unmet today. This is especially true for detection of higher order interactions. Massive testing of SNPs combinations should no longer be a tedious task, but rather a routine operation in a GWAS analysis workflow.

## Conclusion

Currently, no strategy to detect epistasis stands out: all must strike balance between time efficiency and detection power. However, different techniques are available to reduce running times. Some authors improved time efficiency through parallelization of their strategies, e.g., random forests, ant colony optimization and approaches based on computational evolution. Other authors implemented versions of their software programs which use graphic processing units (GPU) instead of traditional central processing units (CPU).

### Conflict of interest statement

The authors declare that the research was conducted in the absence of any commercial or financial relationships that could be construed as a potential conflict of interest.
